# General practitioner experiences using a low back pain management booklet aiming to decrease non-indicated imaging for low back pain

**DOI:** 10.1186/s43058-022-00317-y

**Published:** 2022-06-28

**Authors:** Hazel J. Jenkins, Niamh A. Moloney, Simon D. French, Chris G. Maher, Blake F. Dear, John S. Magnussen, Mark J. Hancock

**Affiliations:** 1 Department of Health Professions, Faculty of Medicine, Health and Human Sciences, Macquarie University, Sydney, Australia; 2 Department of Chiropractic, Faculty of Medicine, Health and Human Sciences, Macquarie University, Level 2, 75 Talavera Rd, Sydney, 2109 Australia; 3 Department of Exercise Sciences, University of Auckland, Auckland, New Zealand; 4 Sydney School of Public Health, Faculty of Medicine and Health, University of Sydney, Sydney, Australia; 5 Institute for Musculoskeletal Health, Sydney, Australia; 6 Department of Psychology, Faculty of Medicine, Health and Human Sciences, Macquarie University, Sydney, Australia; 7 Department of Clinical Medicine, Faculty of Medicine Health and Human Sciences, Macquarie University, Sydney, Australia

**Keywords:** Low back pain, Diagnostic imaging, General practitioners, Patient education, Implementation science, Feasibility studies

## Abstract

**Background:**

Imaging is overused in the management of low back pain, resulting in overdiagnosis, increased healthcare utilisation, and increased costs. Few effective interventions to decrease inappropriate use have been developed and have typically not been developed using behaviour change theory. An intervention to reduce non-indicated imaging for low back pain was developed using behavioural change theory, incorporating a novel low back pain management booklet to facilitate patient education and reassurance. The aim of this study was to assess the adoption and feasibility of use of the developed intervention within clinical practice and to determine appropriate implementation strategies to address identified barriers to use.

**Methods:**

Fourteen general medical practitioners were recruited and trained to use the booklet with low back pain patients over a minimum 5-month period. Quantitative data on use of the booklet were collected and analysed descriptively. Qualitative data on use of the booklet and training session were collected in general medical practitioner interviews and thematically analysed. Barriers to use were identified and mapped to suitable implementation strategies using the Behaviour Change Wheel.

**Results:**

Practitioners used the booklet with 73 patients. The booklet was used with 63% of patients presenting with low back pain. Facilitators for using the booklet included patient’s requesting imaging and lower practitioner confidence in managing low back pain. Barriers included accessible storage and remembering to use the booklet. Implementation strategies were identified to increase adoption and feasibility of use, including development of a digital version of the booklet.

**Conclusions:**

General medical practitioners reported that the low back pain management booklet and training were useful for clinical practice, particularly with patients requesting imaging. Barriers to use were identified and implementation strategies to address these barriers will be incorporated into future effectiveness studies. This study forms one of a series of studies to thoroughly develop and test an intervention to reduce non-indicated imaging for low back pain; a successful intervention would decrease healthcare costs and improve patient management.

**Supplementary Information:**

The online version contains supplementary material available at 10.1186/s43058-022-00317-y.

Contributions to the literature

- The adoption and feasibility of use of a unique intervention (including a low back pain management booklet) designed to reduce non-indicated imaging for low back pain were assessed.
- General medical practitioners found the booklet most useful when patients were requesting non-indicated imaging or needed more reassurance.
- Key barriers to use included a lack of storage space and remembering to use the booklet.
- Implementation strategies to improve adoption and feasibility of use of the intervention were developed using the Behaviour Change Wheel and the Theoretical Domains Framework, a novel approach in the management of low back pain.

## Background

Imaging is overused in the management of low back pain (LBP), with approximately one third of imaging referrals inconsistent with clinical guidelines [1]. Imaging is indicated when there is suspicion of serious underlying pathology such as infection or cancer but does not generally improve outcomes for patients with non-specific LBP [2, 3]. Overuse of imaging may lead to inappropriate diagnoses, further unnecessary investigation or treatment, and unnecessary radiation exposure [2–5]. Decreasing non-indicated imaging for LBP in general practice is challenging: few effective interventions have been demonstrated to date [6] and few have been developed using behaviour change theory [7].

An intervention was recently developed [8] to help general medical practitioners reduce non-indicated imaging for LBP. The research team identified clinician and patient behaviours within a clinical consult which may lead to an overuse of imaging for LBP and developed an intervention to address these behaviours using the Behaviour Change Wheel [9] and the Theoretical Domains Framework [10] (Additional file 1). The intervention included clinician training and provision of a LBP management booklet designed to be used during clinical interactions. The novel booklet (available online at https://tinyurl.com/lowbackpaineducation [11]) was purpose-designed by the research team to address behaviours leading to overuse of imaging. The booklet can be used to screen the patient for indicators for imaging, educate and reassure the patient about LBP and the need for imaging, and provide a customised patient management plan.

Initial feedback on the booklet was sought from general medical practitioners and health care consumers [8]; however, they did not have any actual experience using the booklet in clinical practice. General feedback on the acceptability and appropriateness of the booklet was positive, although some practitioners raised implementation concerns related to the potential adoption and feasibility of use of the booklet in clinical practice. Before effectiveness studies are undertaken, it is important to assess the use of the booklet in clinical practice to ensure that implementation is achievable.

Therefore, the aim of this study was to assess the adoption and feasibility of use of the developed intervention within clinical practice and to determine appropriate implementation strategies to address identified barriers to use.

## Methods

General medical practitioners from metropolitan Sydney, Australia, were asked to use the intervention within their clinical practice. Semi-structured interviews were used to explore practitioners’ experiences using the intervention and alignment with the proposed theoretical model (Additional file 1). Barriers to use were described using the Theoretical Domains Framework. Implementation strategies to address identified barriers were developed using the Behaviour Change Wheel. Ethics approval was granted by Macquarie University Human Research Ethics Committee, reference number: 5201600298. The study and intervention were reported in accordance with the consolidated criteria for reporting qualitative studies (COREQ) and template for intervention description and replication (TIDieR) checklists (Additional files 2 and 3).

### Practitioner recruitment

To enable diversity in participant characteristics that may impact use of the intervention (socioeconomic region of practice location, further education or special interest in LBP, years in clinical practice, sex), purposive sampling of general medical practitioners was performed between May to October 2017. Practitioners or practices known to the research team, and those recommended by participating practitioners, were approached. To be eligible, practitioners needed to self-report that they were currently seeing patients with LBP to ensure that practitioners would have sufficient opportunity to use the intervention during the study period. Based on the sample size needed for a related qualitative study on the development of the booklet [8], we estimated a minimum of 10 practitioners of adequate diversity would be required for this study to generate sufficient data to meaningfully explore the research aims [12]. Further practitioners were recruited as required to ensure practitioners reflected the desired diversity.

### Study procedure

Practitioners attended a 20-min face-to-face training session with one of the research team (HJ) to instruct them in the study aims and requirements and to deliver the training session developed for the intervention (Additional file 4). Demographic information and beliefs about the usefulness of imaging for LBP were obtained (Additional file 5).

The study period ran from May 2017 to April 2018. In the training session, practitioners were asked to use the booklet with patients presenting with LBP who they considered were not indicated for imaging and complete a de-identified record sheet of all low back patients. Recorded data included if and how the booklet was used, imaging referral, LBP characteristics, and suspicion of underlying pathology (Additional file 6).

At the conclusion of the study period, practitioners participated in a 15-min audio-recorded semi-structured interview with one of the researchers (HJ). Questions related to practitioner behaviour were developed using the theoretical domains framework [13, 14] (Additional file 5). Practitioners were given an AUD$60 gift voucher for their time in attending the training session and participating in the end of study interview.

### Quantitative data analysis

Data from the de-identified patient record sheets (Additional file 6) were used to assess how general medical practitioners used the booklet, including the proportion of patients the LBP management booklet was used with; characteristics of patients with whom the booklet was used (e.g. previous history of back pain or imaging); and how the booklet was used with each patient (e.g. within the consult, as a handout).

### Qualitative data analysis

Interviews were transcribed by one researcher (HJ) and imported into NVivo qualitative data analysis software, QSR International Pty Ltd. Version 12, 2018 for analysis. Coding was performed for each study aim prior to performing thematic analysis [15]. To assess how the booklet was used in clinical practice, initial coding was aligned with the theoretical model underpinning the development of the intervention (Additional file 1). Aims relating to clinician behaviour were initially coded using the domains outlined in the Theoretical Domains Framework [13]. Coded data were collated based on similarity, leading to the generation of common themes related to each study aim.

Two researchers (HJ and NM), both with prior experience in coding and using the theoretical domains framework, independently coded three interviews. Coding was compared and discussed, and sufficient consistency was observed between the two researchers after two rounds of discussion to allow one researcher (HJ) to code the remaining interviews. Themes were initially developed by HJ, before discussion with MH, NM, and SF to reach consensus. The resultant themes were then sent to all authors for overall discussion and final consensus.

### Mapping of implementation strategies to address identified barriers

The Behaviour Change Wheel [16] was used to map the identified barriers to using the booklet to appropriate implementation strategies designed to increase use of the booklet in clinical practice. In this process, integration of the COM-B model (Capability, Opportunity, Motivation – Behaviour) and the 14 behavioural domains in the theoretical domains framework were used to map identified barriers to specific behavioural domains requiring change. Appropriate behavioural change techniques and implementation strategies were selected to address each domain, with techniques/strategies prioritised according to the APEASE criteria (Affordability, Practicability, Effectiveness and cost-effectiveness, Acceptability, Side-effects and safety, Equity) [16] and suggestions from practitioners to improve implementation of the intervention. Proctor’s specifications were used to specifically define, describe, and justify the implementation strategies selected [17]. One researcher (HJ) performed the initial mapping, which was then discussed and finalised with the research team.

## Results

Twenty-one general medical practitioners were approached to participate. Of these, four (19%) declined as they either reported that they infrequently saw patients with LBP (*N* = 3) or did not want to participate (*N* = 1). Of the 17 practitioners who participated in the study, 14 (82%) completed the interview at the end of the study. Practitioners had on average 16 years clinical experience, tended to have completed continuing education in LBP (64%), and did not agree that imaging is useful for LBP (Table 1).

**Table 1:** General medical practitioner characteristics (*N* = 14)

Female: *N* (%)	8 (57)
Years in clinical practice: mean (SD)	16.6 (10)
Continuing education in low back pain in last 2 years: *N* (%)	9 (64)
Special interest in low back pain	2 (14)
Agreement with ‘Imaging of the lumbar spine is useful in the workup of patients with acute low back pain’: *N* (%)	Completely disagree: 8 (57) Disagree: 6 (43)
Agreement with ‘I am likely to order imaging for acute low back pain’: *N* (%)	Completely disagree: 13 (93) Disagree: 1 (7)
Socioeconomic area of practice location: *N* (%)	Low: 2 (14) Medium: 5(36) High: 7 (50)

### Adoption and use of the intervention by general medical practitioners

Practitioners participated in the study for between five to 11 months (mean, SD: 8.4, 2.2), depending on their date of recruitment into the study. All practitioners attended the training session. Practitioners used the booklet with 73 LBP patients (mean, SD: 5.2, 4.1). Practitioners reported seeing 99 patients with LBP during the study period; however, only seven of the practitioners (50%) reported completing the patient record form for all LBP patients. For these practitioners, they used the booklet on average with 62.5% (SD: 38.2; 95% CI: 27.2–97.8) of patients presenting with LBP, with reported use ranging from 14.3 to 100.0%. The other practitioners only completed the patient record form when they used the booklet, so percentage of use could not be calculated. During the interviews, these practitioners estimated using the booklet, on average, with 13.6% (SD: 13.1; 95% CI: 1.5–25.7) of patients presenting with LBP, with use ranging from 0.0 to 50.0%.

The patient record form was fully completed for 71% of patients (52/73) with whom practitioners used the booklet, with partial data available for the rest. Most patients had previous episodes of LBP (68%), but the current episode duration was less than 2 weeks (58%). Serious pathology was rarely suspected (7%) and 11% were referred for imaging (Table 2). When practitioners used the booklet, they commonly provided the booklet to patients to take home (56/60, 93.3%; 95% CI: 84.1–97.4), with the patient management plan completed as directed in the training session for most of these patients (52/56, 92.9%; 95% CI: 83.0–97.2). For the remaining patients, practitioners discussed the booklet with patients who subsequently declined to take it home (4/60, 6.7%; 95% CI: 2.6–15.9).

**Table 2:** Characteristics of patients with whom practitioners used the booklet

History of previous episodes of low back pain (%; 95% CI)	39/57 (68.4; 55.5–79.0)
Duration of pain (%; 95% CI)	Less than 2 weeks: 30/52 (57.7; 44.2–70.1) 2–12 weeks: 10/52 (19.2; 13.7–36.1) More than 12 weeks: 12/52 (23.1; 13.7–36.1)
Previously seen a health care provider for current episode of low back pain (%; 95% CI)	30/57 (52.6; 39.3–65.0)
Previous imaging for low back pain (%; 95% CI)	16/57 (28.1; 18.1–40.8)
Clinical suspicion of serious pathology n/N (%; 95% CI)	4/57 (7.0; 2.8–16.7)
Referred for imaging at current visit (%; 95% CI)	6/57 (10.5; 4.9–21.1)

Themes related to how practitioners used the booklet are presented in Table 3. Practitioners did not always discuss the booklet with patients during the consult if they were running short of time or felt they could adequately reassure and educate the patient without the booklet. However, when appropriate, they provided the booklet for the patient to read at home to present further information or reinforce messages delivered during the visit.

**Table 3:** Themes related to ‘How general medical practitioners used the booklet’

Theme	Quotes
Used as designed throughout the consult to (1) show patients why they do not require imaging, (2) demonstrate key educational messages, and (3) provide a customised patient management plan	“I go through it [the booklet] together with them [patients], so I actually use it as an educational tool” (GP2) “I like the diagrams that are in there [decision tree at beginning] that I can sort of go through and say, well you don’t have all these symptoms, so you don’t need any imaging” (GP2) “Yes, that’s not bad [to have somewhere to write patient management] because you’re not giving them necessarily a prescription for prescription drugs, so it doesn’t hurt to write something down, some instructions, and when to come back in for review” (GP8)
Used at the end of the consult only, by customising the management plan and providing it to the patient	“Mostly at the end of the consultation, I’d talk to them about it all and then at the end I’d remember to use it [the booklet], and go through it then and fill in some information” (GP9)
No customisation, given to the patient as a hand-out to read at home at the end of the consult only	“If I thought that someone didn’t need imaging, I simply, towards the end of the consult, gave it [the booklet] to them. I gave it to them to take and read, and in our practice, there was a follow-up appointment made at the time, and at that time we discussed the content of the book“ (GP5)
Used throughout consult to discuss the key messages, but not customised or given to the patient	“Whilst I did go through it [the booklet] with a few patients who were half-interested in looking at it, they didn’t want to take it away, they just thought that they didn’t want the material but were happy just to talk about it” (GP6)

Most practitioners (11/14) reported that they found the booklet useful and would be likely to continue using it in the future, particularly with specific patients, that is, those that requested imaging or required more reassurance or information about their LBP.

“I genuinely think it’s [the booklet] really useful and I’ll continue to use it” (GP10)

“Not everybody like this [continue to use the booklet moving forward], but those who are not easy to convince so those who need more information about back pain who aren’t aware what’s, yes.” (GP13)

One practitioner did not use the booklet during the study, and two practitioners reported that they would be unlikely to continue to use the booklet. These three practitioners reported that they already felt confident that patients would follow their advice without additional resources.

“I think it [the booklet] would be reassuring for lots of clinicians but for me personally I think I can communicate my confidence to the patient and I might be wrong but I feel they’re OK with me just explaining why they don’t need anything” (GP1)

“I’m pretty confident that I don’t need to do the imaging in the first place, so I don’t know whether it [using the booklet] makes a tremendous difference for me really” (GP7)

### Feasibility of using the intervention in clinical practice

Themes relating to barriers and facilitators impacting on general medical practitioners’ use of the LBP management booklet are presented in Table 4. Key barriers included the ability to conveniently store and remember to use the booklet and a lack of time during the consult. A digital version of the booklet was suggested as more convenient to store and remember to use. Facilitators included the ease of use of the booklet and the usefulness of the booklet to help educate and reassure the patient in a time efficient manner. The request for imaging by the patient acted as a reminder to use the booklet.

**Table 4:** Themes related to ‘Barriers and facilitators impacting use of the intervention’

Theme	Facilitator or barrier	Quotes
Storage location and remembering to use the booklet	Facilitator: Storing the booklet in a visible location with convenient access	“Yes I did find the booklet OK to use, and because it was somewhere where I can reach it, it was good” (GP2)
	Facilitator: Patient requesting imaging	“I only tend to think of it [the booklet] when people ask for imaging, so that’s probably a positive” (GP10)
	Barrier: Nowhere to store the booklet with good visibility or convenient access	“In offices you just lose pieces of paper and little booklets and all of the rest. You don’t have room to store everything” (GP4)
	Barrier: Forgetting to use the booklet	“I only used the one and I think that’s probably not the booklet, but because it’s difficult to remember” (GP1)
Clinician having the necessary knowledge/skills to use the booklet	Facilitator: Training or clinician prior knowledge was sufficient to use the booklet	“I think it [the training] was absolutely fine, the booklet’s quite self-explanatory, it’s quite clearly laid out so that was fine” (GP1)
	Barrier: Some points were missed in the training session, and the booklet was not used completely	“Yes, I think I missed a few points [in training] so that’s what I failed to explain fully to my patients” (GP14)
Perceived usefulness of the booklet within a consult	Facilitator: The information in the booklet is appropriate, useful for patient education, and helps to reinforce practitioner confidence and recommendations	“I actually found the booklet really comprehensive. I found it really helpful [to reduce unnecessary imaging], so I don’t think you need, I mean I wouldn’t use other things” (GP2) “It [the booklet] probably backs me up, makes me feel more confident, and I think I’ve got some research backing me up and then I can counter it [patient request for imaging], and I can say well look there’s this and they’ve done this, and they’ve looked at this, and if you’re worried then this can be our plan” (GP3) “I think the booklet was, for me, a quick way of explaining the rationale behind not imaging, and the patient seemed to appreciate this to a greater depth when given the booklet” (GP5) “It [the booklet] also helped me, remind me of a few things which I forget sometimes because I can’t necessarily always remember all these things or sometimes I just focus more on one thing or the other” (GP7) “I think giving people written data, you know like a written pamphlet, gives a bit more credibility to what you say, so you can educate people about not needing imaging” (GP11)
	Facilitator: The booklet was used because the clinician felt the patient required more education or reassurance	“I think for instance I felt [in the patients that did use the booklet with] there was an expectation that was either voiced or implied of imaging, and so to sort of counter that view the booklet was handy” (GP5) “I think if you did have someone who was quite adamant to want imaging it [the booklet] would be then more useful for those certain patients” (GP6)
	Barrier: Booklet was not needed as current clinician method of managing clinical consults sufficient	“I think it [the booklet] would be reassuring for lots of clinicians but for me personally I think I can communicate my confidence to the patient and I might be wrong but I feel they’re OK with me just explaining why they don’t need anything” (GP1) “I’m pretty confident that I don’t need to do the imaging in the first place, so I don’t know whether it [using the booklet] makes a tremendous difference for me really” (GP7)
	Barrier: Clinician felt the patient did not require more education or reassurance	“Not everybody comes and asks for an X-ray, some of them understand it’s muscular not underlying bone pathology there you know” (GP13)
	Barrier: Low back pain an uncommon presentation for the clinician	“I might see a back pain patient you know, maybe only once a fortnight because I don’t have that big throughput” (GP3)
Time efficiency of using the booklet in a consult	Facilitator: Use of the booklet improved time efficiency in the consult	“I think also at least in a couple of cases [when used the booklet] that I recall, I was very much pushed for time. It’s handy to say, here it is, have a read” (GP5)
	Barrier: Not enough time in a consult to use additional resources	“The time factor [why didn’t use the booklet with other patients], because if lots of patients are waiting, if you don’t have a lot of time, then I didn’t go into this much detail” (GP13)
	Barrier: Using the booklet took additional time in the consult	“I mean it [using the booklet] did add time for me. I could imagine that there could be ways to do it that it wouldn’t, but that’s just not how I, I suppose, talk to people” (GP9)
Perceived receptiveness of the patient to receiving the booklet	Facilitator: Clinician felt the patient would be receptive to receiving the booklet	“Yes they [the patients] liked it [the booklet], I think patients always like to go away with something, so yes I think they liked it” (GP9)
	Barrier: Clinician felt the patient would not be receptive to receiving the booklet	“I guess it [the booklet] helps reinforce the message for people who are accepting the message, but I think the people that really have come in with an agenda and you can’t sway them, the booklet’s not going to sway” (GP4) “Whilst I did go through it [the booklet] with a few patients who were half-interested in looking at it, they didn’t want to take it away” (GP6)

### Mapping of barriers to implementation strategies

The mapping of the identified barriers to implementation strategies is presented in Table 5 with definitions of the implementation strategies outlined in Additional file 7. The key behavioural domains addressed were those of psychological capability and reflective motivation. Additional implementation strategies selected in this process included the following: development of a digital version of the booklet to allow for easy storage, hardcopy booklets available for patients in the reception area, reminders to use the booklet through the practice management software, audit and feedback of imaging referral behaviour to clinicians, and selection of a local opinion leader to champion use of the booklet.

**Table 5:** Mapping barriers to using the intervention to implementation strategies

Barrier	COM-B/TDF domain	Behavioural change techniques	Implementation strategy (EPOC taxonomy)	Implementation strategy (detail)
Nowhere to store the booklet with good visibility or convenient access	Physical opportunity/environmental	Adding objects to the environment	Educational materials	Patient education booklet provided in both digital and hardcopy formats
			Environment	Areas identified or created to place booklets (waiting room, office space)
Forgetting to use the booklet	Psychological capability/memory	Adding objects to the environment	Educational materials	Patient education booklet provided in both digital and hardcopy formats
		Prompts/cues	Reminders	Automatic reminders to use booklet through practice management software
		Information about social and environmental consequences	Educational outreach visit	Strategies to remember to use the booklet discussed in the individualised training session for the clinician
Some points were missed in the training session, and the booklet was not used completely	Psychological capability/knowledge	Information about social and environmental consequences	Educational outreach visit	Individualised training session for clinician with discussion of key points and modelling use of the booklet
			Educational materials	Training resources provided for future clinician reference (low back pain guidelines, training video and sheets to use the booklet)
Booklet was not needed as current clinician method of managing clinical consults sufficient	Reflective motivation/beliefs about capabilities	Feedback on outcomes of behaviour	Audit and feedback	Low back imaging referral audit, provided to the clinician (individual and population data) to show current imaging referral behaviour
		Feedback on outcomes of behaviour	Educational outreach visit	Individualised training session for clinician with discussion of how the booklet may help in different scenarios
		Credible source	Local opinion leader	Champion within each clinic to encourage active engagement with decreasing non-indicated imaging for low back pain
Clinician felt the patient did not require more education or reassurance	Reflective motivation/beliefs about consequences	Information about social and environmental consequences	Educational outreach visit	Individualised training session for clinician with discussion of patient beliefs and need for reassurance
Not enough time in a consult to use additional resources	Physical capability/physical skills Reflective motivation/Beliefs about consequences	Instruction on how to perform a behaviour Demonstration of the behaviour	Educational outreach visit	Individualised training session for clinician with modelling of how to use the booklet and educate the patient within a standard consult
Clinician felt the patient would not be receptive to receiving the booklet	Reflective motivation/beliefs about consequences	Information about social and environmental consequences	Educational outreach visit	Individualised training session for clinician with discussion of patient receptiveness for educational resources
		Credible source	Local opinion leader	Champion within each clinic to encourage active engagement with decreasing non-indicated imaging for low back pain

## Discussion

This study found that general medical practitioners varied in their use of the developed intervention to reduce non-indicated imaging. Low users of the booklet were more likely to be confident in their management of LBP and reported not needing additional resources. Higher use was reported when patients requested non-indicated imaging or needed more reassurance. The booklet was feasible to use in clinical practice; however, important barriers to use were identified, including available storage and remembering to use the booklet. A digital version of the booklet was strongly favoured by all practitioners.

Strengths of this study included the use of both quantitative and qualitative methods to assess the adoption and feasibility of use of the intervention in clinical practice. Quantitative data showed variable use of the booklet by general medical practitioners and qualitative analysis used the Theoretical Domains Framework to identify and explore barriers and facilitators influencing use. Implementation strategies to address identified barriers were selected using the Behaviour Change Wheel and described using Proctor’s specifications.

A limitation of this research was the lack of feedback from patients regarding their experience in receiving the booklet. Future research would benefit from exploring patient feedback to assess how useful they found the booklet. The use of the booklet varied between practitioners and could not be accurately measured due to incomplete data from general medical practitioners; however, qualitative responses allowed us to explore the barriers limiting use of the booklet and address these for future implementation. Importantly, practitioners tended to remember to use the booklet with patients who requested imaging or needed more education or reassurance, thus, using the booklet in the cases where it is needed.

Study generalisability and the relatively small sample size of practitioners needs to be considered as a possible limitation. This was an exploratory study, and it is possible that with broader sampling or longer interviews, additional barriers and subsequent implementation strategies may have been identified. Sampling of practitioners was performed to achieve diversity in socioeconomic region of practice location, further education or special interest in LBP, years in clinical practice, and sex. Diversity was not achieved in beliefs about the need for imaging, with all practitioners reporting that imaging is not typically useful in the management of acute LBP. However, previous research has shown that whilst medical practitioners commonly disagree about imaging being important for LBP management [18], they still frequently order imaging. Barriers such as patient pressure for imaging and limited time in a consult (which this intervention was designed to directly address [8]) are thought to be the main drivers of imaging overuse.

The results of this study will be used to further inform the development and implementation of an intervention to reduce non-indicated imaging for LBP in general medical practice. The identified implementation strategies to increase intervention use will be incorporated into the planned studies to assess the effectiveness of the intervention in clinical practice.

## Conclusion

General medical practitioners had variable adoption of a LBP management booklet in clinical practice. Low use was more common in practitioners who were confident in their ability to educate and reassure patients with LBP. Practitioners were more likely to use the booklet if patients requested imaging or required more reassurance about their LBP. Barriers impacting the use of the intervention were identified and strategies to increase use will be incorporated into future implementation measures. This study is one part of a series designed to develop and test an intervention to reduce non-indicated imaging for LBP; a successful intervention would decrease healthcare costs and improve patient management.

## Supplementary Information

**Additional file 1.** Theoretical model of the effect of the low back pain management booklet on known barriers to reducing non-indicated imaging for low back pain.

**Additional file 2.** COREQ checklist.

**Additional file 3.** TIDieR checklist.

**Additional file 4.** Outline of the general medical practitioner training session.

**Additional file 5.** General medical practitioner baseline questionnaire and outline of semi-structured interview questions.

**Additional file 6.** Patient record sheet.

**Additional file 7. **Defining the implementation strategies using Proctor’s specification.

## Data Availability

The datasets used and/or analysed during the current study are available from the corresponding author on reasonable request.

## References

[1] Jenkins HJ, Downie AS, Maher CG, Moloney NA, Magnussen JS, Hancock MJ (2018). Imaging for low back pain: is clinical use consistent with guidelines? A systematic review and meta-analysis. Spine J.

[2] Chou R, Qaseem A, Owens D, Shekelle P (2011). Diagnostic imaging for low back pain: advice for high-value health care from the American College of Physicians. Ann Intern Med.

[3] Maher C, Underwood M, Buchbinder R (2017). Non-specific low back pain. Lancet.

[4] Chou R, Deyo RA, Jarvik JG (2012). Appropriate use of lumbar imaging for evaluation of low back pain. Radiol Clin North Am.

[5] Lemmers G, van Lankveld W, Westert G, van der Wees P, Staal J (2019). Imaging versus no imaging for low back pain: a systematic review, measuring costs, healthcare utilization and absence from work. Eur Spine J.

[6] Jenkins HJ, Hancock MJ, French SD, Maher CG, Engel RM, Magnussen JS (2015). Effectiveness of interventions designed to reduce the use of imaging for low-back pain: a systematic review. Can Med Assoc J.

[7] Hodder RK, Wolfenden L, Kamper SJ, Lee H, Williams A, O’Brien KM (2016). Developing implementation science to improve the translation of research to address low back pain: a critical review. Best Pract Res Clin Rheumatol.

[8] Jenkins HJ, Moloney NA, French SD, Maher CG, Dear BF, Magnussen JS (2018). Using behaviour change theory and preliminary testing to develop an implementation intervention to reduce imaging for low back pain. BMC Health Serv Res.

[9] Michie S, van Stralen MM, West R (2011). The behaviour change wheel: a new method for characterising and designing behaviour change interventions. Implement Sci.

[10] Atkins L, Francis J, Islam R, O’Connor D, Patey A, Ivers N (2017). A guide to using the Theoretical Domains Framework of behaviour change to investigate implementation problems. Implement Sci.

[11] Jenkins H (2020). Low back pain management booklet Macquarie Univeristy: Macquarie University.

[12] Braun V, Clarke V (2021). To saturate or not to saturate? Questioning data saturation as a useful concept for thematic analysis and sample-size rationales. Qual Res Sport Exerc Health.

[13] Huijg JM, Gebhardt WA, Crone MR, Dusseldorp E, Presseau J (2014). Discriminant content validity of a theoretical domains framework questionnaire for use in implementation research. Implement Sci.

[14] Michie S, Johnston M, Abraham C, Lawton R, Parker D, Walker A (2005). Making psychological theory useful for implementing evidence based practice: a consensus approach. Qual Saf Health Care.

[15] Braun V, Clarke V (2006). Using thematic analysis in psychology. Qual Res Psychol.

[16] Michie S, Atkins L, West R (2014). The behaviour change wheel. A guide to designing interventions.

[17] Proctor EK, Powell BJ, McMillen JC (2013). Implementation strategies: recommendations for specifying and reporting. Implement Sci.

[18] Buchbinder R, Staples M, Jolley D (2009). Doctors with a special interest in back pain have poorer knowledge about how to treat back pain. Spine (Philadelphia, Pa 1976).

