# Supplementary material for: General practitioner experiences using a low back pain management booklet aiming to decrease non-indicated imaging for low back pain
Source: Implement Sci Commun. 2022 Jun 28;3:71. doi: 10.1186/s43058-022-00317-y (PMC9238090; doi:10.1186/s43058-022-00317-y)
Supplement: Supplementary file 1 — Additional file 1. Theoretical model of the effect of the low back pain management booklet on known barriers to reducing non-indicated imaging for low back pain. [file 43058_2022_317_MOESM1_ESM.docx]

Additional file 1: Theoretical model of the effect of the low back pain management booklet on known barriers to reducing non-indicated imaging for low back pain


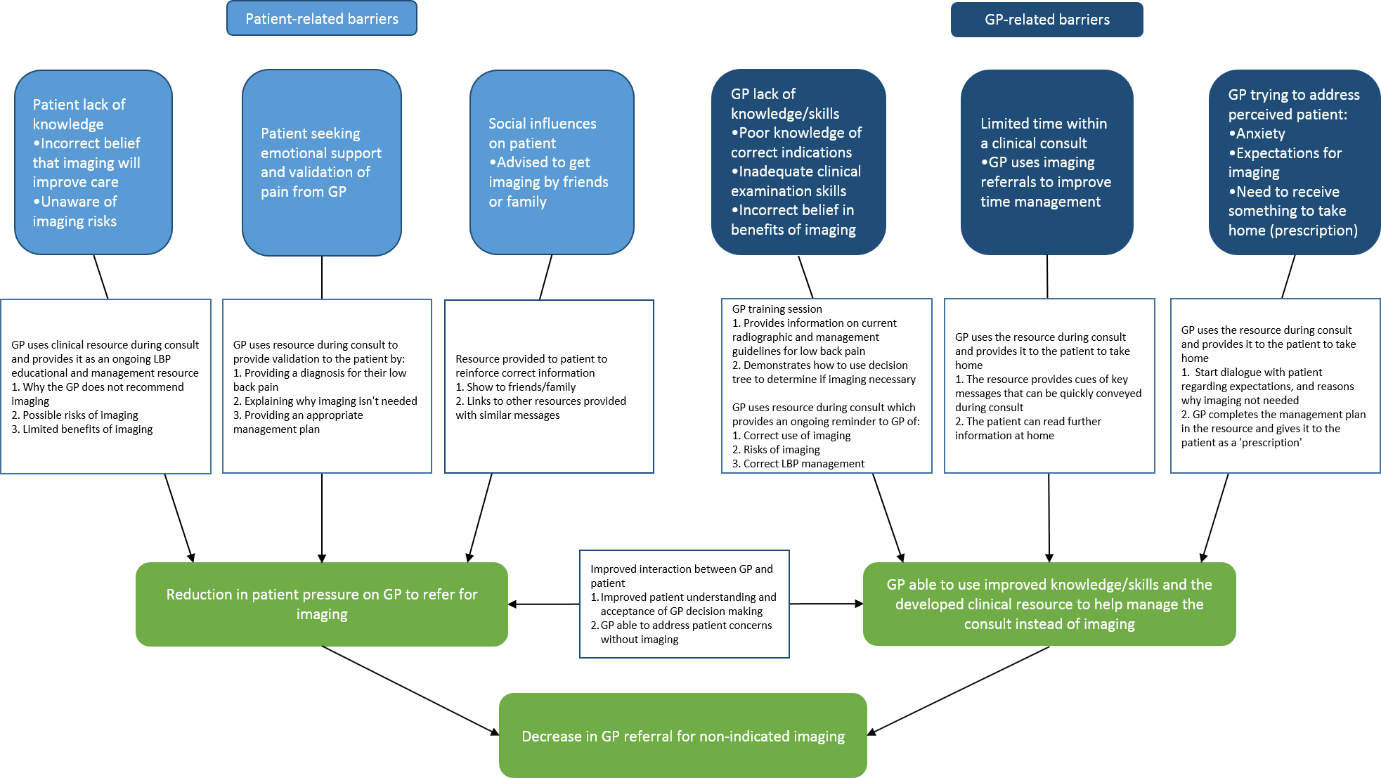


This figure has been republished with consent from Figure 3 in ‘Using behaviour change theory and preliminary testing to develop an implementation intervention to reduce imaging for low back pain’ (8): a concept map of how the intervention was designed to target identified barriers to appropriate use of imaging for low back pain.
