# Supplementary material for: General practitioner experiences using a low back pain management booklet aiming to decrease non-indicated imaging for low back pain
Source: Implement Sci Commun. 2022 Jun 28;3:71. doi: 10.1186/s43058-022-00317-y (PMC9238090; doi:10.1186/s43058-022-00317-y)
Supplement: Supplementary file 4 — Additional file 4. Outline of the general medical practitioner training session. [file 43058_2022_317_MOESM4_ESM.docx]

**Additional file 4: General medical practitioner training session**

The general medical practitoner training is a 20 minute face to face session with a trained facilitator (either a low back pain researcher involved in the study or a facilitator trained by the research team)

The following items will be discussed during the training session:

1. Introduction

- The patient education booklet is a novel educational tool, designed to provide patient education and reassurance, and ultimately decrease the inappropriate use of imaging in the management of low back pain
- The booklet is flexible in its use and how you decide to use it will depend on your clinical judgement
- During this session I will show you how the booklet has been designed to be used and why this will be helpful in clinical practice

2. Appropriate use of imaging in the management of low back pain

- Explain why imaging, when not clinically indicated, is a problem, and why we are trying to reduce this
- Discuss indications for imaging, and the appropriate diagnosis and management of low back pain
- Provide relevant low back pain publications. Highlight imaging guidelines in Maher et al. (Maher *et al.*, 2017), and summary of best evidence in LBP management in Qaseem et al. (Qaseem *et al.*, 2017)

3. Reason for booklet development and the benefits of use

- Provide GP information sheet for ongoing reference
- Outline the intended goals of using the booklet
- Explain why the booklet is different/novel and give reasons for inclusion of each of the elements
  - Decision tree (clinical decision support)
  - Information about low back pain (key educational messages)
  - Information about limitations of imaging (key educational messages)
  - Self-management advice (key educational messages)
  - Management plan
  - When to return/what to do next
  - Further resources
- Outline potential benefits of use
  - Time-efficient way to ensure you provide all necessary information to the patient
  - Aids to help explain to the patient why imaging is not necessary
  - Management plan – giving the patient the booklet as a ‘prescription’ instead of an imaging referral
  - Giving the patient more information about what to do next if symptoms don’t resolve, and other reputable resources
  - Patient can use the booklet as an ongoing resource

4. Demonstration of use of the booklet by the training facilitator

- Demonstrate the suggested use of the booklet
- Emphasise how the booklet can be used time efficiently
- Emphasise the importance of individualising the booklet for the patients

5. Explain which patients might be most appropriate, and which may not respond as well

6. Suggest storage options for the booklet; Explain why a booklet was used rather than an electronic option

7. Ask for any questions

References:

Maher, C., Underwood, M. & Buchbinder, R. (2017) Non-specific low back pain. *The Lancet*, **389**, 736-747.

Qaseem, A., Wilt, T.J., McLean, R.M. & Forciea, M.A. (2017) Noninvasive treatments for acute, subacute, and chronic low back pain: a clinical practice guideline from the American College of Physicians. *Annals of Internal Medicine*, **166**, 514-530.
