# Supplementary material for: General practitioner experiences using a low back pain management booklet aiming to decrease non-indicated imaging for low back pain
Source: Implement Sci Commun. 2022 Jun 28;3:71. doi: 10.1186/s43058-022-00317-y (PMC9238090; doi:10.1186/s43058-022-00317-y)
Supplement: Supplementary file 5 — Additional file 5. General medical practitioner baseline questionnaire and outline of semi-structured interview questions. [file 43058_2022_317_MOESM5_ESM.docx]

**Additional file 5: General medical practitioner baseline questionnaire and interview transcript**

**Baseline Questionnaire**

Code: _______________________

1. Sex: ❒ Male

❒ Female

2. Year of graduation from medical degree: ________________

3. Number of years practicing as a general practitioner: ________________

4. Do you have a special interest in back pain as a general practitioner?

❒ Yes

❒ No

5. Have you done any continuing education in back pain in the last 2 years?

❒ Yes

❒ No

6. We are interested in what you think about imaging for low back pain as a general practitioner. Please indicate your views below by circling the appropriate number on the scale.
1=COMPLETELY DISAGREE 5= COMPLETELY AGREE

COMPLETELY DISAGREE

COMPLETELY AGREE

| Imaging of the lumbar spine is useful in the workup of patients with acute low back pain | 1 | 2 | 3 | 4 | 5 |
| --- | --- | --- | --- | --- | --- |
| I am likely to order imaging for acute low back pain | 1 | 2 | 3 | 4 | 5 |

**Semi-structured Interview Guide**

Questions 1-8 below were asked to all participants. The subsequent probes were used only as required to explore the participants’ responses further.

*Introduction to participant:*

I’d like to ask you some questions about the patient education booklet that you have recently used in clinical practice. There are no right or wrong answers and we are very much interested in your feedback and suggestions for improvement.

Do I have your permission to use de-identified quotes from this interview in publication?

*1. What was your experience in using the booklet in clinical practice*

Possible additional probes:

“How did you use the booklet with patients?”

“Did you use the booklet routinely with patients presenting with simple low back pain or did you pick particular patients? If so, why?” (TDF: memory, attention and decision processes)

“What are your feelings about using this booklet in clinical practice?” (TDF: emotion)

“How many (or what proportion of) low back pain patients did you use the booklet with?” (TDF: behavioural regulation)

*2. “What is your plan in using this booklet with patients with LBP, moving forward*

Possible additional probes:

“How would you continue to use the booklet?” (TDF: goals)

“For the next 10 patients with simple LBP, how many do you intend to use this booklet with?” (TDF: intention)

*3. How practical did you find incorporating the booklet into your consults?*

Possible additional probes:

“Were you able to integrate the booklet into your consults? How did you do this/Why weren’t you able to?” (TDF: skills)

“Did you remember to use this booklet? Why/why not?” (TDF: memory, attention and decision processes)

“Were you able to use the booklet in a time efficient manner? Why/why not?” (TDF: environmental context and resources)

“Where did you place the booklet in your office?” (TDF: environmental context and resources)

“How did you find using this resource as a booklet? Would an online or electronic option be preferable?” (TDF: environmental context and resources)

“Did you feel you had the necessary knowledge and skills to be able to use the booklet in clinical practice?” (TDF: knowledge, skills)

*4. How did you find the training session you received in using the booklet?*

Possible additional probes:

“Was the training you received in using the booklet at an appropriate level? Why/why not?” (TDF: skills, knowledge)

“Did the training help you feel confident in using the booklet?” (TDF: beliefs about capabilities, skills)

*5. “What were your experiences in using the booklet with your patients?”*

Possible additional probes:

“Did you find patient expectations influenced your use of the booklet?” (TDF: social influences)

“How receptive do you think patients were to receiving this booklet?” (TDF: beliefs about consequences)

“How do you think using the booklet would affect the amount of pressure given from patients to refer for imaging?” (TDF: beliefs about consequences)

*6. Do you think that using the booklet helped you to manage patients with LBP without imaging? Why/why not?*

Possible additional probes:

“How did using the booklet affect your decision making process when determining whether to refer a patient with LBP for imaging?” (TDF: memory, attention and decision processes)

“Did using the booklet change your confidence in your ability to manage patients without imaging? Why/why not?” (TDF: beliefs about capabilities)

“Did using the booklet improve your patient communication/reassurance, or your ability to resist patient pressure for imaging?” (TDF: skills)

“Did using the booklet help reduce patient pressure for imaging?” (TDF: beliefs about consequences)

“When you used the booklet, were there circumstances that you still ended up referring for imaging? If so, why?” (TDF: behavioural regulation)

*7. Do you think the booklet is a useful tool for clinical practice? Why/why not?*

Possible additional probes:

“Do you think this booklet, its content and format, is appropriate to use in clinical practice?” (TDF: environmental context and resources)

*8. What suggestions would you make to help improve the booklet or its integration into clinical practice*

Possible additional probes:

“Is there anything you can think of that would make the booklet more appropriate or acceptable to you?” (TDF: environmental context and resources)

“Is there anything you can think of that would help you to use this booklet?” (TDF: reinforcement)

“How would you suggest that the booklet should be rolled out for use in clinical practice?”

“Do you have any suggestions to improve the training session?”

“Could the training be given in a different format (information sheet, online module etc.)?”
