# Supplementary figures and images for: General practitioner experiences using a low back pain management booklet aiming to decrease non-indicated imaging for low back pain
Source: Implement Sci Commun. 2022 Jun 28;3:71. doi: 10.1186/s43058-022-00317-y (PMC9238090; doi:10.1186/s43058-022-00317-y)

Additional file 6: Patient record sheet


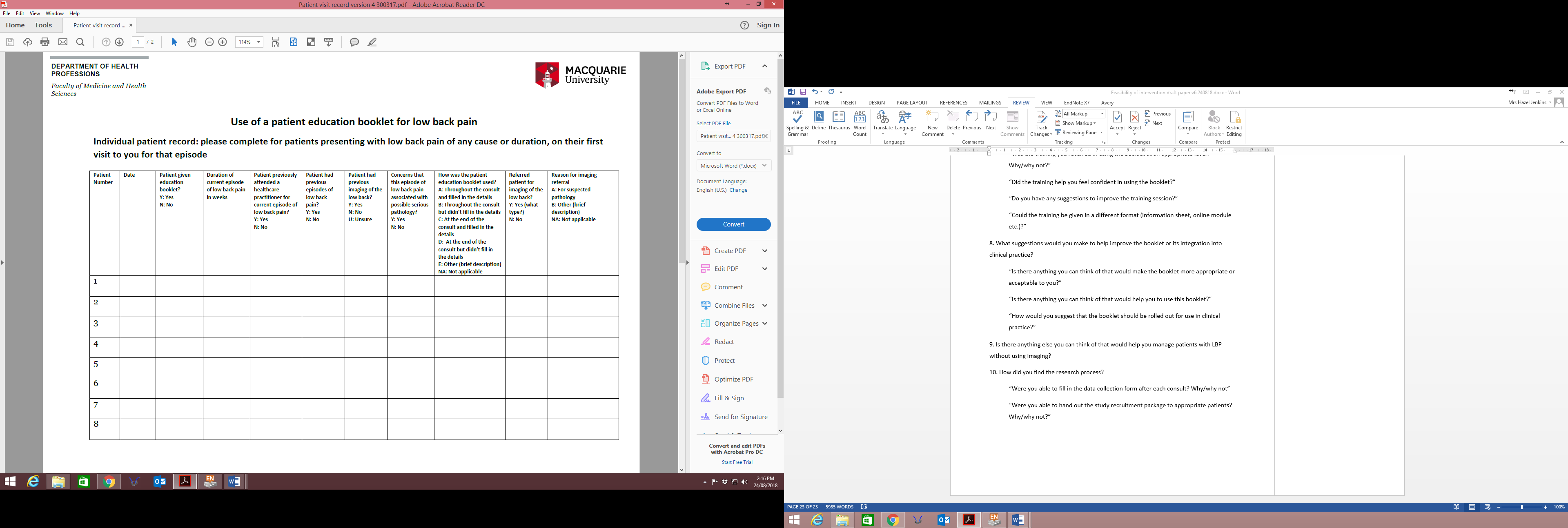

Supplement: Supplementary file 6 — Additional file 6. Patient record sheet. [file 43058_2022_317_MOESM6_ESM.docx]
