# Supplementary material for: General practitioner experiences using a low back pain management booklet aiming to decrease non-indicated imaging for low back pain
Source: Implement Sci Commun. 2022 Jun 28;3:71. doi: 10.1186/s43058-022-00317-y (PMC9238090; doi:10.1186/s43058-022-00317-y)
Supplement: Supplementary file 7 — Additional file 7. Defining the implementation strategies using Proctor’s specification. [file 43058_2022_317_MOESM7_ESM.docx]

Additional file 7: Defining the implementation strategies using Proctor’s specifications

| **Implementation strategy (EPOC taxonomy)** | **Definition** | **Actor** | **Action** | **Action target** | **Temporality** | **Dose** | **Implementation outcome affected** | **Justification** |
| --- | --- | --- | --- | --- | --- | --- | --- | --- |
| Educational materials | 1. Patient education booklet (hardcopy and digital)  2. Training resources (low back pain guidelines, training video and sheets to use the booklet) | Implementation facilitator | 1. Provide booklets to clinic in hardcopy form, arrange for digital versions to be installed on computers  2. Provide training resources | 1. Clinicians – booklets available as needed  2. Clinicians - to complement or remind them of training | 1. With initial training  2. At training session | 1. Re-supply hardcopy booklets as required  2. Provided once. Clinicians can watch training video as required for reminder | 1. Adoption of booklet; Feasibility of using booklet  2. Adoption of booklet; Fidelity of use of booklet. | 1. Providing the booklet and ensuring ease of access will enable clinicians to use (and remember to use) the booklet more easily.  2. Providing training resources will allow clinicians to further educate themselves and use video to remind themselves of correct use of the booklet |
| Environment | Areas identified or created to place booklets (waiting room, office space) | Implementation facilitator and clinic staff | Identify suitable locations for booklet storage and accessibility for both clinician and patient access (waiting room) | Clinicians and patients – to be able to access the booklet when needed | At initial training session | Initial, then ongoing to ensure areas are kept clear for booklet storage, are stocked with booklets | Adoption of booklet; Feasibility of using booklet | Providing the booklet and ensuring ease of access will enable clinicians to use (and remember to use) the booklet more easily. Patients can access in the waiting rooms and bring in to the clinician if interested |
| Reminders | Automatic reminders to use booklet through practice management software | IT assistance | Integrate reminders to use the booklet into the practice management system | Clinicians - to provide reminders to use booklet when low back pain patients present | Prior to implementation and training session | As required to check reminders occurring as programmed | Adoption of booklet; Feasibility of using booklet | Regular reminders through the computer to use the booklet when low back pain patients present will increase adoption and feasibility of use |
| Educational outreach visit | Individualised training session for clinicians | Implementation facilitator | Provide training session | Clinicians - to train them in how to use the booklet in a time-efficient manner, explain why it was designed and what it is intended to address and model how to use the booklet | At start of implementation | Single 30 min face to face individualised training session | Acceptability and appropriateness of booklet, Adoption of booklet, Feasibility and fidelity of use of booklet | Clinicians will be educated on why the booklet was developed, why it is appropriate for them and how the elements in the booklet have been designed to aid patient education. They will be trained in the use of the booklet and shown strategies to use it time efficiently, increasing adoption and feasibility of use. Use of the booklet will be modelled to increase fidelity of use. |
| Audit and feedback | Low back imaging referral audit, provided to clinicians (individual and population data) | Implementation facilitator | Perform audit of imaging use and summarise individual and population imaging use data | Clinicians - to allow them to reflect on their imaging use | Prior to implementation and training session | Ongoing every 6 months (with previous 6 months data) | Adoption of booklet | Imaging use data will be provided against population averages and ideal use to demonstrate whether tools such as the booklet are required. |
| Local opinion leader | Champion within each clinician’s clinic | Clinician champion in clinic | Encourage and promote use of the booklet | Clinicians - to encourage them to use the booklet and be active in reducing imaging use | Prior to implementation and training session | Ongoing encouragement | Acceptability and appropriateness of the booklet, adoption of the booklet | A clinician champion will be selected in the clinic to help lead the other clinicians and to reinforce why the booklet would be beneficial and the need to reduce imaging rates |
